# Label-free quantitative phosphoproteomics with novel pairwise abundance normalization reveals synergistic RAS and CIP2A signaling

**DOI:** 10.1038/srep13099

**Published:** 2015-08-17

**Authors:** Otto Kauko, Teemu Daniel Laajala, Mikael Jumppanen, Petteri Hintsanen, Veronika Suni, Pekka Haapaniemi, Garry Corthals, Tero Aittokallio, Jukka Westermarck, Susumu Y. Imanishi

**Affiliations:** 1 Turku Centre for Biotechnology, University of Turku and Åbo Akademi University, Tykistokatu 6, FI-20520 Turku, Finland; 2 Department of Pathology, University of Turku, FI-20520 Turku, Finland; 3 Turku Doctoral Program of Biomedical Sciences (TuBS), Turku, Finland; 4 Department of Mathematics and Statistics, University of Turku, FI-20014 Turku, Finland; 5 Drug Research Doctoral Programme (DRDP), Turku, Finland; 6 Institute for Molecular Medicine Finland, Tukholmankatu 8, FI-00290 Helsinki, Finland; 7 Turku Centre for Computer Science, FI-20520 Turku, Finland; 8 Van ‘t Hoff Institute for Molecular Sciences (HIMS), University of Amsterdam, Science Park 904, 1098 XH Amsterdam, The Netherlands; 9 Faculty of Pharmacy, Meijo University, Yagotoyama 150, Tempaku, Nagoya 468-8503, Japan

## Abstract

Hyperactivated RAS drives progression of many human malignancies. However, oncogenic activity of RAS is dependent on simultaneous inactivation of protein phosphatase 2A (PP2A) activity. Although PP2A is known to regulate some of the RAS effector pathways, it has not been systematically assessed how these proteins functionally interact. Here we have analyzed phosphoproteomes regulated by either RAS or PP2A, by phosphopeptide enrichment followed by mass-spectrometry-based label-free quantification. To allow data normalization in situations where depletion of RAS or PP2A inhibitor CIP2A causes a large uni-directional change in the phosphopeptide abundance, we developed a novel normalization strategy, named pairwise normalization. This normalization is based on adjusting phosphopeptide abundances measured before and after the enrichment. The superior performance of the pairwise normalization was verified by various independent methods. Additionally, we demonstrate how the selected normalization method influences the downstream analyses and interpretation of pathway activities. Consequently, bioinformatics analysis of RAS and CIP2A regulated phosphoproteomes revealed a significant overlap in their functional pathways. This is most likely biologically meaningful as we observed a synergistic survival effect between CIP2A and RAS expression as well as KRAS activating mutations in TCGA pan-cancer data set, and synergistic relationship between CIP2A and KRAS depletion in colony growth assays.

Cancer associated changes commonly alter the activity of kinase signaling pathways, many of which are potentially druggable^1^,^2^. RAS family GTPases H-RAS, K-RAS, and N-RAS are prominent oncogenes that function as key upstream regulators of multiple cancer-associated pathways^3^. RAS genes frequently undergo mutational activation in cancer^4^ and in some cancers these mutations have a complementary distribution with the other activating mutations of the major downstream serine/threonine kinase pathways, PI3K/AKT and MAPK/ERK^5^. However, phosphorylation levels of proteins, and therefore activities of signaling pathways, are determined by the balance of phosphatase and kinase activity^6^. Protein phosphatase 2A (PP2A) either alone or together with PP1 dephosphorylates the majority of all serine and threonine phosphorylated proteins^7^,^8^. PP2A activity is commonly inhibited in cancer cells by overexpression of endogenous inhibitor proteins^9^, inactivating mutations and deletions of certain subunits^7^,^10^, and post-translational modifications of the catalytic subunit^11^. Cancerous inhibitor of PP2A (CIP2A) is an endogenous inhibitor of PP2A with oncogenic properties^12^. It is overexpressed and correlates with disease progression in wide variety of human cancers^13^. Importantly, it has been shown that PP2A antagonizes oncogenic activity of hyperactivated RAS in cellular transformation^14^,^15^,^16^,^17^ and in cell cycle control^18^, and furthermore, PP2A inhibition by CIP2A overexpression synergizes with the RAS-mediated transformation^12^,^19^. However, even though PP2A is known to regulate several RAS effector kinase pathways^3^ (Fig. 1a), it has not been systematically assessed how RAS activity and PP2A inhibition functionally cooperate in regulation of protein phosphorylation.

Phosphoproteomics analysis allows for site-specific identification and quantification of a large number of phosphoproteins^20^,^21^,^22^,^23^,^24^,^25^,^26^,^27^. A general workflow consists of proteolytic digestion of proteins and then selective enrichment for phosphopeptides prior to their analysis by liquid chromatography-tandem mass spectrometry (LC-MS/MS). Optimized sample preparation procedures and recent MS instruments enable hundreds or thousands of phosphopeptide identifications from the single measurement. Quantification of global phosphoproteome has often been performed by using stable isotope labeling techniques, such as a metabolic labeling method SILAC (stable isotope labeling by amino acids in cell culture; typically 2–3 samples per analysis) and a chemical labeling method iTRAQ (isobaric tag for relative and absolute quantitation; typically 4–8 samples per analysis)^21^,^24^,^28^,^29^. Once samples are labeled and mixed, the abundance ratios of phosphopeptides are maintained throughout the sample processing and measurement, which leads to improved accuracy in quantification. Recently, an alternative label-free quantification method, particularly based on peptide abundance (precursor ion abundance), has been introduced in the global phosphoproteomics field^30^,^31^,^32^,^33^. Although label-free quantification requires careful experimental design to maintain reproducibility, it can be used to avoid some of the drawbacks of labeling methods, including labeling reagent cost, inefficient labeling, difficulty in low abundance peptide analysis, and the limitation of sample number^23^. Label-free approaches provide benefits especially for large-scale analyses, e.g. experiments done with various treatment conditions, or clinical screening applications. For instance, de Graaf *et al*. have reported a label-free temporal phosphoproteomics study on Jurkat T cells that consisted of >100 LC-MS/MS data to be compared^34^.

One of the concerns related to label-free quantification is how to accurately normalize measured phosphopeptide abundance. Thus far, global centering normalization methods such as those based on the mean/total abundance and median abundance ratio have most commonly been used^31^,^33^,^34^,^35^,^36^,^37^,^38^. These methods can be applied if the majority of the phosphorylations can be assumed unaltered across the samples. However, when a large-scale change in the global protein phosphorylation occurs (Fig. 1b), e.g. during mitosis^39^ or in response to EGF stimulation of serum starved HeLa cells^20^ (both SILAC-based studies), the assumptions of the centering normalization do not hold anymore. In fact, it is hard to justify those assumptions in many phosphoproteomics studies since dynamic regulations of kinases and/or phosphatases are expected to be seen there. Also from a technical point of view, due to variation introduced in the phosphopeptide enrichment step, in addition to the fluctuating nanoflow LC and ionization conditions, the phosphorylation profile before the enrichment is difficult to predict. Analysis of those samples would require alternative normalization methods such as spiking in known quantities of phosphoproteins/phosphopeptides^30^,^40^.

Here, we have studied global phosphorylation changes in HeLa cells when PP2A is activated by depleting CIP2A or inhibited by okadaic acid (OA) treatment. OA is a potent small molecule PP2A inhibitor that is commonly used to interrogate PP2A’s functions although it inhibits also other serine/threonine phosphatases, exhibiting approximately 100-fold selectivity to PP2A/PP4/PP6 over PP1/PP3^41^,^42^. Due to the large number of PP2A targets, we expected a global dephosphorylation to occur when PP2A is activated and global upregulation when PP2A is inhibited. Additionally, we depleted the RAS proteins, due to the suggested functional antagonism between PP2A and RAS in regulation of several pathways^43^. The expected effects on global protein phosphorylation caused by these perturbations are depicted in Fig. 1a. By studying these model samples, we demonstrate the importance of selecting an appropriate normalization method in label-free quantitative phosphoproteomics, as well as propose a novel approach to achieve accurate quantification. Importantly, this approach enabled the monitoring of true phosphoproteome dynamics, which revealed novel insights into the synergy between PP2A inhibition and RAS in cancer cells.

## Results

### Identification and quantification of proteins and phosphorylations by LC-MS/MS analysis

As model samples for label-free quantitative phosphoproteomics, we used HeLa cells treated with CIP2A siRNA, RAS siRNA, and OA as well as with control siRNA (control 1), in biological triplicates. We used a cocktail siRNA targeting H-, K-, and N-RAS for the reason that in HeLa cells the different RAS isoforms do not exhibit specificity towards the downstream AKT and ERK pathways, and efficient downregulation of these pathways has been shown to require targeting more than one RAS isoform^44^. The experimental workflow is shown in Fig. 2a. Cell lysates (1 mg protein each) were spiked in with a phosphoprotein bovine α-casein (10 μg), and then digested with trypsin in parallel. The majority of the digests (99% v/v) were enriched for phosphopeptides by TiO_2_ affinity chromatography sequentially. The samples with and without the enrichment were subjected to LC-MS/MS analysis (Q Exactive, Thermo Fisher Scientific). The lysates of the same control samples were processed again on different days as a technical replicate (control 2), and analyzed together with the above samples. Mascot database searching (Matrix Science) was performed for identifying peptides and proteins, and phosphorylation site localization was validated using phosphoRS^45^. We also performed SpectraST searching against a simulated phosphopeptide spectral library (SimSpectraST searching), which is highly sensitive for the site-specific identification of phosphopeptides covered by the library^46^. The combination of these orthogonal methods improved the confidence of the identifications. When score cutoffs for a false-localization rate (FLR) of 1% were applied (i.e. high confidence phosphosites), the site disagreement by Mascot and SimSpectraST on shared sequence identifications was improved from 12% to 1.4%, as expected (Supplementary Table 1). Label-free quantification was performed using Progenesis software (Nonlinear Dynamics). Peptide ion features were aligned, detected, and then quantified based on precursor ion abundance. Based on the chromatographic data alignment, it is possible to measure all the detectable peptides even when peptides are unidentified in some samples. Phosphosites (combinations) were quantified by summing the feature abundance, where low confidence site features were excluded from quantification of high confidence sites. The numbers of identifications and quantifications are summarized in Table 1. From the TiO_2_-enriched samples, we identified a total of 4,519 unique phosphopeptides, at a false-discovery rate (FDR) of 0.18% using the target-decoy strategy at a phosphopeptide spectral match level (Supplementary Table 1). Out of those, 3,073 unique phosphopeptides with 2,621 phosphosite combinations were quantified based on 4,026 ion features (Supplementary Tables 2 and 3), which included 2,911 phosphosites on 1,255 proteins (2,051 high confidence sites on 1,067 proteins). From the non-enriched digests, we identified 16,344 unique peptides at a peptide spectral match level FDR of 0.15%, which resulted in quantification of 14,015 unique peptides and 2,567 proteins based on 16,922 ion features (Supplementary Tables 4 and 5). Also, 68 unique phosphopeptides were quantified without the TiO_2_-enrichment, of which 52 could be used for a newly developed normalization method (Fig. 2b) as described below.

### Quantitative measurement of phosphopeptides with different normalization methods

TiO_2_ enrichment is regarded as a major source of variation for label-free quantification, and indeed it constituted a large part of variance in our platform (Supplementary Fig. 1). Therefore, an appropriate normalization of phosphopeptide abundance needs to be applied. By using the dataset obtained from the TiO_2_-enriched samples, we investigated how different normalization methods affect the outcomes of label-free phosphoproteomics studies. First, we tested the commonly used normalization methods, including centering normalizations (global median ratio centering and quantile-based normalization, henceforth global centering and quantile centering, respectively) and the normalization by spiked internal standards (α-casein phosphopeptides). The fold change distributions of phosphopeptide ion features were monitored for the CIP2A, RAS, and OA samples compared to the control 1 samples. In the non-normalized data we observed mostly upregulations compared to the control 1 samples (Fig. 3a,b). As expected, the normalizations had a large impact on the distributions in terms of shifting their mean/median values (Fig. 3a). These shifts were reflected in the ratio of up- and down-regulated phosphorylations (differentially regulated phosphosites compared to the control 1 samples; t-test, p < 0.01) (Fig. 3b). The global centering and the quantile centering normalizations of the data yielded similar ratios of the regulated phosphorylations across all the treatments (50–63% upregulation). In contrast, the casein normalization failed to correct the unlikely result of pronounced upregulation in all samples in the non-normalized data (Supplementary Fig. 2a). Variations in spiking α-casein, presumably due to the limited accuracy in protein concentration measurement of cell lysates, seem to have contributed to this trend (Supplementary Fig. 2b). Thereby we conclude that use of any of the tested normalization methods do not reveal the expected profound upregulation of protein phosphorylation by OA treatment and downregulation by CIP2A and RAS depletions.

### Pairwise normalization developed for label-free quantitative phosphoproteomics

**Figure 1:**
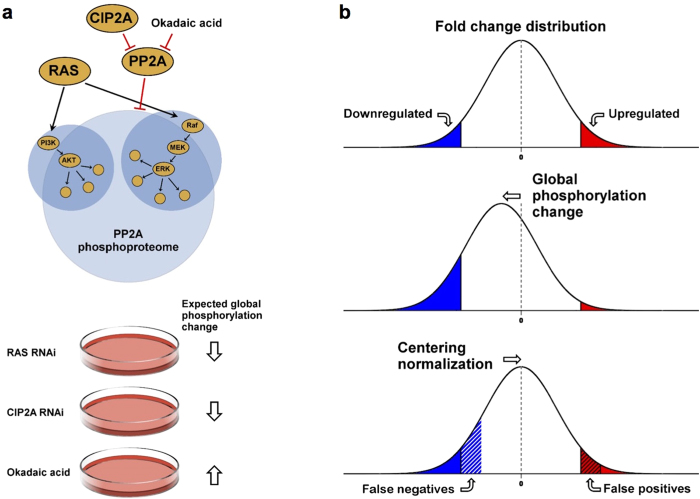
A schematic effect of a normalization bias caused by manipulation of RAS and PP2A phosphoproteomes. (**a**) Protein phosphatase 2A (PP2A) participates in the regulation of a large part of phosphoproteome, including major serine/threonine kinases AKT and ERK that are also key downstream effectors of the RAS oncoproteins. RNAi mediated depletion of RAS, PP2A activation by depletion of CIP2A protein, and PP2A inhibition by OA were used as model perturbations, to study the influence of global phosphorylation changes on the performance of different normalization methods in label-free quantitative phosphoproteomics. (**b**) Centering normalization is often used in quantitative proteomics and phosphoproteomics data (upper panel). However, a global phosphorylation change shifts the distribution of the phosphorylation ratios (middle panel). In such cases, centering leads to normalization bias, which introduces false positive phosphorylations in the opposite direction from the global change and also false negatives in the direction of the global change (lower panel).

**Figure 2:**
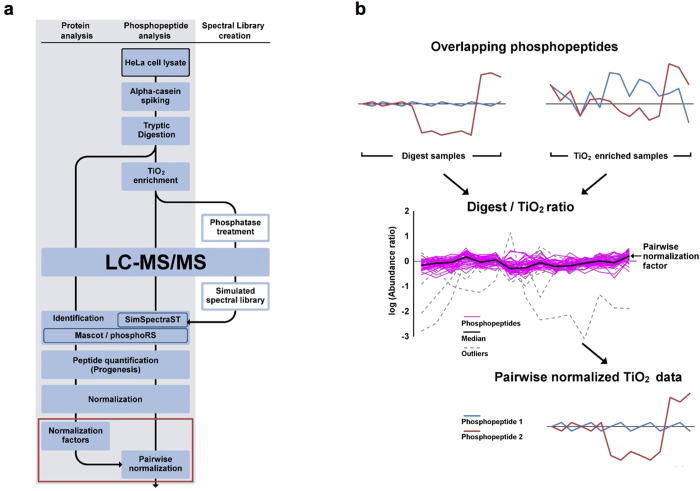
Pairwise normalization developed for label-free quantitative phosphoproteomics. (**a**) HeLa cells with different treatments were subjected to cell lysis, spiking *α*-casein standard, and tryptic digestion. Peptides with and without TiO_2_ phosphopeptide enrichment were analyzed by LC-MS/MS. Peptides were identified by Mascot database search, followed by phosphorylation site validation by phosphoRS. Phosphopeptide identification was supplemented by SimSpectraST spectral library search. Following label-free quantification, peptide abundance was normalized with different methods, including the pairwise normalization for TiO_2_ data developed in this study. (**b**) The principle of the pairwise normalization method. Fifty-two phosphopeptides were quantified in both the non-enriched digests and TiO_2_-enriched samples (i.e. 52 digest-TiO_2_ pairs). Abundance profiles of two hypothetical phosphopeptides are illustrated as examples. An abundance ratio was calculated by pairwise comparison (digest/TiO_2_) for each phosphopeptide. Eleven pairs were excluded as outliers (see the criteria in Supplementary Fig. 3). The median of normalized abundance ratios was then calculated for the remaining 41 pairs and used as a pairwise normalization factor for the TiO_2_ data. The TiO_2_ data were pre-normalized with the global centering method, whereas the digest data were normalized with the global centering or quantile centering method (i.e. global pairwise and quantile pairwise, respectively).

**Figure 3:**
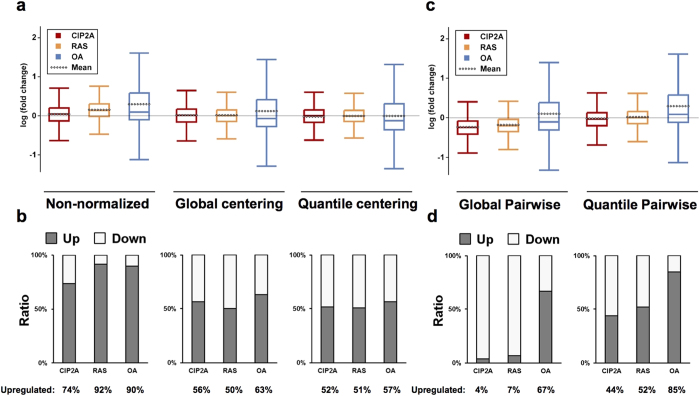
Fold change distributions of phosphorylations after different normalizations. (**a**) Fold changes for each phosphopeptide ion feature was calculated for the CIP2A, RAS, or OA samples compared to the control 1 samples (log-transformed). The abundance of the features was normalized with global centering and quantile centering methods. Median and mean levels are marked with a solid and dashed line on the box plots, respectively, and whiskers represent 1.5 × interquartile range. (**b**) Ratio of up- and down-regulated phosphosites (differentially regulated phosphosites compared to the control 1 samples; t-test, p < 0.01) is shown for both normalization methods and non-normalized data. Abundances of the features with identical protein phosphorylations were summed up for calculating phosphosite abundance. The centering normalizations resulted in similar ratios of up- and downregulated phophosites in contrast to the expected phosphoproteome changes (i.e. increase in protein phosphorylation after OA treatment and dephosphorylation after CIP2A or RAS depletion, refer to Fig. 1a). (**c**) Fold changes of phosphopeptide features and (**d**) ratio of up- and down-regulated phosphosites (t-test, p < 0.01) after pairwise normalizations. Global pairwise normalization of the data resulted in the best agreement with the expected global phosphoproteome changes (see Fig. 1a).

As illustrated in Fig. 1b and also exemplified in Fig. 3, the centering normalization methods may introduce a systematic error into label-free quantitative phosphoproteomics in some cases, and even result in quantification bias. However, as mentioned above, if the assumptions of the centering normalization do not hold, predicting the original phosphoproteome profiles is challenging when phosphopeptides are enriched without labeling. In this study, we rationalized that normalization of TiO_2_-enriched phosphopeptides could be corrected by using phosphopeptides observed prior to the enrichment as reference peptides. As the non-enriched digests are dominated by nonphosphorylated peptides (99.5% of the quantified peptides, see Table 1), their normalization is not significantly influenced by global phosphorylation changes. Therefore, it is expected that phosphopeptide abundance in the non-enriched samples can be more accurately quantified based on the centering normalization than that in the enriched samples. We used phosphopeptides that were quantified both in the non-enriched digests and TiO_2_-enriched samples, and calculated a digest/TiO_2_ abundance ratio for each phosphopeptide after global centering normalization (Fig. 2b). The TiO_2_-enriched data were then normalized using the median of these ratios as a normalization factor. We observed a total of 52 phosphopeptides for this purpose, of which 41 were used for calculating the normalization factor (Fig. 2b). Eleven were excluded as outliers due to not being quantified in every sample or due to having extreme fold changes between samples (Supplementary Fig. 3).

As the proposed strategy is based on pairwise comparison of the same phosphopeptides from non-enriched and TiO_2_-enriched samples, we call this novel method as pairwise normalization method. The pairwise normalization factors were calculated based on two centering normalizations of the non-enriched digest data, i.e. global centering and quantile centering normalizations. These are termed as global pairwise and quantile pairwise normalizations, respectively, and their performance was evaluated. In contrast to the other three normalizations (Fig. 3 and Supplementary Fig. 2), both of the pairwise normalization methods resulted in significantly larger difference between the OA and CIP2A/RAS samples (Supplementary Table 6), with majority of phosphorylations upregulated in the OA samples (global pairwise: 67%, quantile pairwise: 85%) (Fig. 3c,d). Furthermore, the expected downregulation was clearly observed in the CIP2A and RAS samples in the global-pairwise-normalized data (96% and 93%, respectively). Based on these results, the global pairwise normalization conformed best to the original hypothesis illustrated in Fig. 1a.

To challenge our observation, we further looked into the distributions of phosphopeptide feature abundance and fold change ratios. Regardless of the normalization, the fold change distribution in the OA samples was markedly wider than in the CIP2A or RAS samples (Fig. 3a,c). In the global-pairwise-normalized data, this could be attributed to upregulation, often several fold, of a large number of low abundance features in the OA samples, compared to those in the control 1 samples (Supplementary fig. 4a). The abundance distribution change in the CIP2A samples was subtler but a large number of phosphopeptide ions, mainly high abundance ones, were shifted towards the median (Supplementary fig. 4a). These changes resulted in reduced variability in the abundance distributions of phosphopeptide features in the CIP2A, RAS and OA samples than in the control 1 and 2 samples (Supplementary fig. 4b). Although similar changes in the abundance distributions could not be observed in the quantile-centering-normalized data (Supplementary fig. 4a), the fold change distribution in the OA samples still had a marked positive skew and the distinctly increased mean values compared to the median (Supplementary fig. 4c), supporting the observation that the upregulation of a significant portion of the phosphorylations actually occurred in the OA samples.

### Clustering analysis of the samples after different normalizations

**Figure 4:**
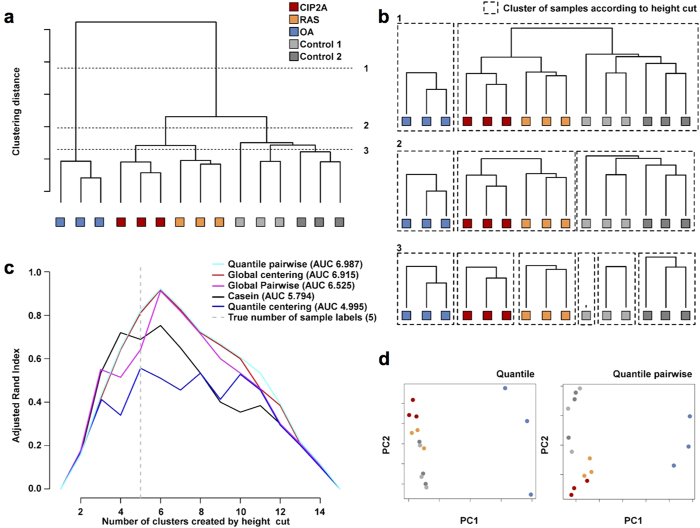
Hierarchical clustering of the samples after different normalizations. (**a**) The log-transformed, normalized phosphosite data was clustered using a variety of distance metrics and clustering strategies. Euclidean distance-based Ward’s minimum variance clustering for the global-pairwise-normalized data is shown here as an example. CIP2A and RAS formed a tight cluster that was clearly separated from OA, and also distinguished from the control sample cluster. (**b**) Various cuts on the clustering distance height were applied (horizontal lines 1, 2 or 3 in panel a) to produce subclusters of different sizes. Here, clustering solutions with 2, 3 or 6 clusters are shown. (**c**) The sample clusters at various height cuts were compared to the original sample groups using the adjusted Rand index computed for each of the 5 normalization methods, and AUC was used to compare between the methods. The AUC values for different clustering parameter combinations are shown in Supplementary Table 7. (**d**) PCA plots for the quantile-centering-normalized and quantile-pairwise-normalized data. Variance among the OA samples led to sub-optimal grouping in the quantile centering normalization (left panel).

Even though the quantitative data normalized with the global pairwise method fits the original hypothesis best, we wanted to further compare the normalization methods by performing a sample clustering on the data in order to study the ability of the normalization methods to distinguish between sample groups. We used a total of 16 combinations of clustering strategies on the 5 versions of the normalized data. Representative clustering for global pairwise normalization is shown in Fig. 4a and the concept of clustering performance evaluation in Fig. 4b. Details are described in materials and methods section. Supplementary Table 7 contains the area under the curve (AUC) values for the adjusted Rand indices from the unsupervised clustering, Out of the tested clustering strategies, the combinations of Euclidean distance or Pearson correlation with Ward clustering resulted in the best classification accuracies. In these analyses, the 5 sample groups were clearly distinguishable with most normalizations and clustering options, but the best performance was obtained with quantile pairwise normalization, followed closely by global centering, global pairwise, and casein normalizations (Fig. 4c). The control samples 1 and 2 clustered close together as expected (Fig. 4a). The CIP2A and RAS samples clustered as well (Fig. 4a), suggesting similarities in their phosphoproteomes.

The relatively poor performance of the quantile centering normalization was partly attributed to the dispersion of the OA samples. Fig. 4d shows the principal component analysis (PCA) plots for the quantile centering and the best performing quantile pairwise normalizations. The relative variance of the three OA samples is much larger in the quantile-centering-normalized data, and additionally the control samples 1 and 2 were less distinguishable from the CIP2A/RAS samples than in the quantile pairwise PCA. The clustering performance was further tested by excluding the OA samples (Supplementary Table 7), which improved the performance of the quantile centering normalization while keeping the order of the normalization methods the same. Overall, the sample groups were well separated with appropriate clustering parameters but the quantile centering normalization was found inferior to the other normalizations in distinguishing the sample groups.

### Western blotting validation of quantitative results obtained with different normalizations

**Figure 5:**
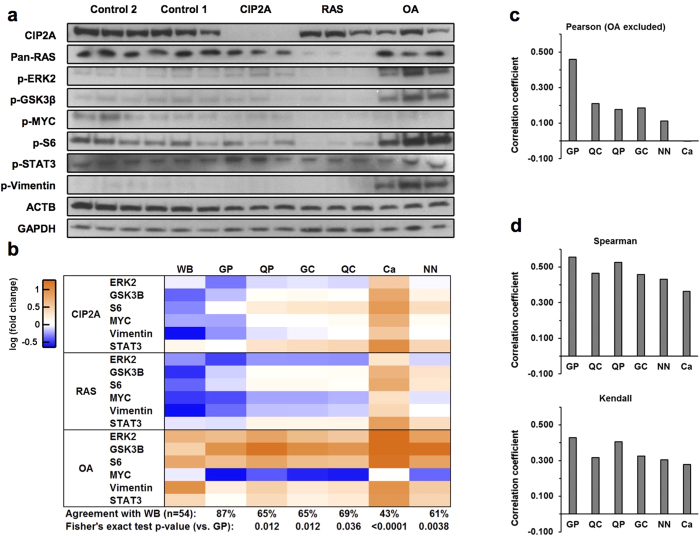
Western blot validation of phosphorylations. (**a**) Western blotting was performed on the cell lysates used for LC-MS/MS analysis. Representative western blots for each antibody are shown. (See Supplementary Fig. 5 for different exposure times). (**b**) Quantitative results of the phosphorylation regulations obtained by western blotting were compared with LC-MS/MS results with different normalizations. Fold-changes (average of triplicates) compared to the control 1 samples are shown. The directions of phosphosite regulations (i.e. up or down) in the CIP2A, RAS, and OA samples (individual replicates) were also compared to the average of control 1 samples. The agreement with western blot was compared between different normalizations using Fisher’s exact test. (**c**) Average correlation coefficients for phosphosites were calculated between the western blotting and LC-MS/MS results on log-transformed data. As the OA samples significantly skewed the data dominating the Pearson’s correlation coefficients, they were excluded from the calculations. Global pairwise normalization led to the highest correlation. (**d**) Spearman’s ρ and Kendall’s τ rank correlation coefficients were also calculated for phosphosites in all samples (i.e. the OA samples included). WB: western blotting, GP: global pairwise, QC: quantile centering, QP: quantile pairwise, GC: global centering, NN: non-normalized, and Ca: casein.

Results above indicate that the newly developed pairwise normalization methods might be able to solve the perceived problems observed when using the centering normalization methods. To confirm the improved performance of the methods, we validated the quantitative results using western blotting. The following seven phosphorylation sites were monitored: ERK2 T185/Y187, GSK3β S9, MYC S62, S6 S235/S236, STAT3 S727, vimentin S56 and AKT S473. Six of these phosphorylation sites were also observed in the LC-MS/MS data and used for investigating the correlation between these two quantification methods (Fig. 5). Representative western blots are shown in Fig. 5a and quantitations are shown in Supplementary Fig. 5 and Supplementary Table 8. Efficient downregulation of CIP2A and RAS were confirmed, and importantly they did not regulate each other (Fig. 5a). ERK and AKT phosphorylations regulated by RAS (Fig. 1a) were also confirmed. ERK2 T185/Y187 was downregulated by RAS depletion and upregulated by OA (Fig. 5a,b). AKT S473 phosphorylation was downregulated by depletion of CIP2A and RAS at a comparable level and upregulated by OA (Supplementary Fig. 5). The phosphorylation changes observed in the western blot analysis are concordant with previous literature: Although participating in the activation of Raf-MEK-ERK pathway, PP2A inhibition has been associated with sustained and amplified ERK activation^47^. PP2A directly dephosphorylates AKT^48^, and CIP2A has been shown to influence AKT phosphorylation^49^. Inactivating S9 phosphorylation of GSK3β has been shown to be dephosphorylated by PP2A^50^. RAS stabilizes MYC via promoting S62 phosphorylation by ERK, and also via inactivating GSK3β through PI3K/AKT pathway^51^. Also CIP2A promotes MYC stability by inhibiting the dephosphorylation of S62^12^. PP2A inhibits^52^, and RAS/ERK signaling promotes, the activity of p70 S6 kinase that is responsible for phosphorylating S6 S235/S236^53^. When the direction of phosphosite regulation (i.e. up or down) was compared, global pairwise normalization exhibited significantly higher level of concordance with western blotting results than the other normalizations (Fig. 5b).

Based on the quantitative results, correlation coefficients between the western blotting and LC-MS/MS data were calculated (Fig. 5c and Supplementary Table 9). In support of their good performance in data normalization observed by the other approaches, both of the pairwise normalization methods had the highest Pearson’s correlation with western blot quantification (Supplementary Table 9). However, the OA treatment induced significant changes at some phosphorylation sites, thus skewing the distribution of quantified intensities despite the log-transformation of the data. To accommodate for this, we repeated the correlation analyses either by excluding the OA samples (Fig. 5c) or by using the nonparametric correlation measures (Fig. 5d). Systematically, global pairwise normalization showed the highest correlations with the western blotting data (Fig. 5c,d). Thus, we conclude that out of the all normalization methods tested in this study, the global pairwise normalization has the superior capacity as an abundance normalization method for analysis of label-free quantitative phosphoproteomics data in conditions in which global changes in protein phosphorylation are expected.

### Pathway analysis using the appropriate normalization methods

**Figure 6:**
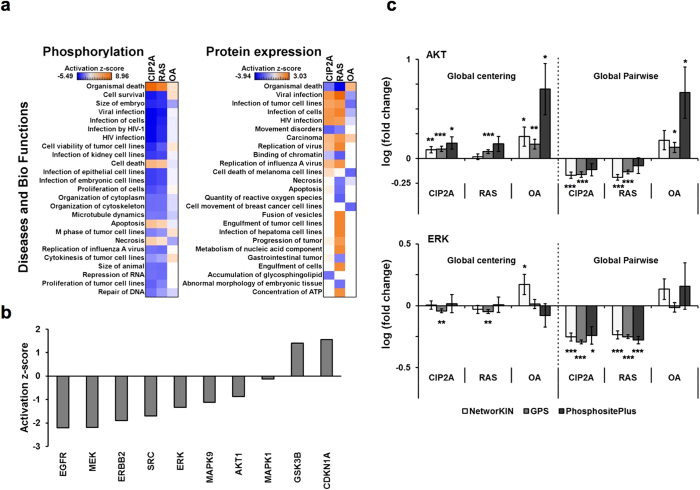
Pathway analysis for protein and phosphorylation regulations. (**a**) The protein and phosphosite fold changes (compared to control 1) were calculated from global-centering-normalized non-enriched data and global-pairwise-normalized TiO_2_ data, respectively. In Ingenuity Pathway Analysis, core analysis was performed for differentially regulated proteins and phosphosites (t-test, p < 0.05), followed by comparison analysis between the CIP2A, RAS, and OA core analyses. The top hits from the category “Diseases and Bio functions” are shown. (**b**) The phosphosite data was filtered for those regulated by both CIP2A and RAS depletions (t-test, p < 0.05), and the core analysis was performed. Upstream regulator analysis restricted to kinases is shown. (**c**) AKT and ERK target sites were predicted by NetworKIN and GPS tools or retrieved from the PhosphositePlus database (see Supplementary Fig. 8). The average fold changes for AKT and ERK target sites are presented for the global-centering (left) and global-pairwise (right) normalized data. The expected regulations of AKT and ERK mediated phosphorylations were clearly observed by global pairwise normalization. The error bars represent standard error of the mean (SEM). The asterisks represent level of statistical significance for up-/down-regulations (one sample t-test, *p < 0.05, **p < 0.01, ***p < 0.001).

Based on the above results, we selected the global pairwise normalization as the appropriate normalization method for label-free quantitative phosphoproteomics. To gain an understanding of the biological processes regulated by CIP2A, RAS, and OA phosphoproteomes, the global-pairwise-normalized data was next subjected to Ingenuity Pathway Analysis (Qiagen). Interestingly, the results from this analysis supported the novel findings (Fig. 4a) that the phosphoproteomes regulated by CIP2A and RAS are involved in highly similar biological functions, including regulation of cell death, survival, and proliferation (Fig. 6a). In contrast, the OA treatment had the opposite effect on several of these functional categories. However, when the non-enriched digest data with global centering normalization was analyzed by pathway analysis, this revealed that at the level of protein expression, CIP2A and RAS have more diverse effects (Fig. 6a), partly due to RAS depletion regulating the expression of a larger number of proteins than CIP2A depletion or OA treatment (Supplementary Fig. 6). Many proteins regulated uniquely by RAS were associated to carbohydrate metabolism and other metabolic pathways (Supplementary Fig. 7).

To identify the key regulators of the common functions of CIP2A and RAS, we performed Ingenuity upstream regulator analysis of the CIP2A-RAS shared phosphoproteome regulation and, strikingly, the suggested upstream kinases were almost solely members of the RAS downstream pathways MAPK/ERK, PI3K/AKT, and MAPK/JNK2, as well as tyrosine kinases functioning upstream of RAS (Fig. 6b)^54^. However, these Ingenuity analyses are designed for expression level data, which raises concerns about its applicability to phosphorylation data. Therefore, we also monitored the phosphorylation changes specifically at ERK and AKT targeted sites. The sites predicted by two tools, NetworKIN^55^ and GPS^56^, as well as the sites curated from literature into PhosphoSitePlus database^57^, were taken into consideration (Supplementary Table 3). The threshold for prediction scores was determined by comparing the predictions to the known target proteins curated from literature (Supplementary Fig. 8). This resulted in 53/150/18 AKT target sites and 60/251/19 ERK targets sites for NetworKIN, GPS, and PhosphositePlus, respectively. In the global-pairwise-normalized data, the average phosphorylation levels at the AKT and ERK sites were downregulated by CIP2A and RAS depletion (AKT: 1.2–1.6 fold, ERK: 1.7–2.0 fold) and upregulated by OA (AKT 1.3–4.6 fold, ERK 1.4 fold except for GPS)(Fig. 6c). This is again consistent with the expected results illustrated in Fig. 1a. The same trend was not observed with the global centering normalization (Fig. 6c). Interestingly, the phosphorylation levels at the ERK and AKT target sites in the global-pairwise-normalized data correlated between the CIP2A and RAS samples (Supplementary Fig. 9 and Supplementary Table 10) suggesting that the same AKT and ERK targets are under the regulation of CIP2A and RAS. These results support the idea that applying different normalizations can leads to distinct biological conclusions in the phosphoproteomics studies.

### CIP2A and KRAS regulate cancer cell growth and determine patient survival synergistically

**Figure 7:**
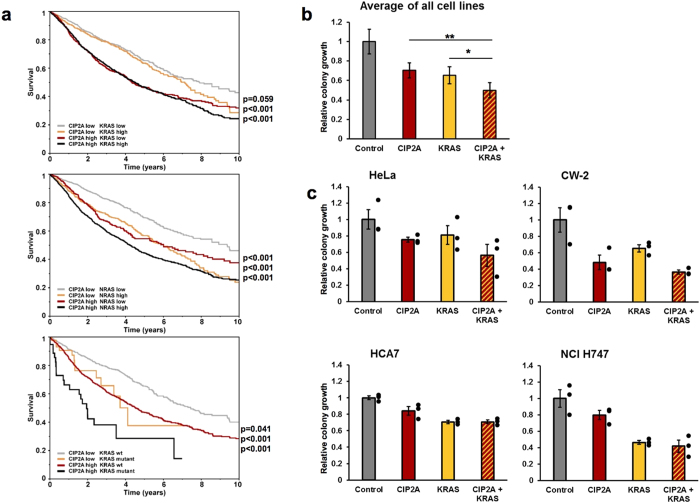
CIP2A and KRAS show synergy in TCGA data and in colony formation assay. (**a**) The Kaplan-Meier plot of TCGA pan-cancer survival profiles. The patients were split into groups by pan-cancer normalized gene-expression data for KRAS, NRAS and CIP2A (downregulated < 0, upregulated ≥ 0), and by KRAS mutational status. Combination of low CIP2A expression with non-mutated or low expression of RAS was associated with best survival. Log-rank test was used for comparing the survival distributions between the group with the best survival and the other groups. (**b**) Colony formation assay following CIP2A and KRAS depletions as well as CIP2A + KRAS co-depletion. Assay was performed 3 times using different siRNAs for CIP2A in HeLa, CW-2, HCA7, and NCI H747 cell lines. The average result is shown. See also Supplementary Fig. 10 for the triple-RAS depletion. (**c**) Averages of 3 colony formation experiments for each cell line. The error bars represent SEM. The asterisks represent level of statistical significance (t-test, *p = 0.0162, **p = 0.0012).

To assess whether the overlapping pathway regulation by CIP2A and RAS is biologically meaningful, we analyzed The Cancer Genome Atlas (TCGA) pan-cancer data for potential interactions between CIP2A expression and RAS isoform expression/mutations on patient survival analysis. The survival analysis was limited to 10-year follow-up time. High expression of CIP2A, NRAS, and to lesser extent KRAS was associated with poor prognosis in TCGA pan-cancer data set (Fig. 7a). Furthermore, we observed a synergistic survival effect between CIP2A and KRAS or NRAS expression. The combination of high CIP2A and high K- or N-RAS expression was associated with the worst survival and the combination of low CIP2A and low K- or N-RAS expression with the best survival (Fig. 7a). We did not see clear synergy between CIP2A and HRAS expression. Although overexpression of RAS proteins has been shown to contribute to cancer^58^,^59^, the oncogenic RAS signaling is commonly activated by mutation. Therefore, we also analyzed the relationship between CIP2A expression and RAS mutations. Mutational data and gene expression data (pan-cancer normalized) are available for a limited number of patients only; nevertheless, KRAS activating mutations exhibited similar synergistic relationship with CIP2A expression levels as did high KRAS expression (Fig. 7a). The poor survival of KRAS mutants is partly explained by enrichment of lung adenocarcinoma in these groups. However, the cancer type does not account for the survival effect of CIP2A. In contrast to the observed survival, the weighted average of expected 5 year survival rate was higher for CIP2A high/KRAS mutant than for CIP2A low/KRAS mutant patient group (Supplementary Table 11), suggesting that high CIP2A expression is an indicator of poor prognosis in KRAS mutant cancers. Taken together, the combination of low CIP2A expression with low expression or wild-type KRAS resulted in a survival advantage in the TCGA data.

In order to examine the potential causal relationship we performed a series of colony formation experiments on HeLa as well as CW-2, HCA7, and NCI-H747 colorectal cancer cell lines. Three different siRNAs were used for CIP2A with no apparent difference in colony formation efficiency. On average, depletion of both CIP2A and KRAS impaired the colony formation more efficiently than depletion of either alone (Fig. 7b). This synergistic relationship between CIP2A and KRAS depletion was most evident in HeLa and CW-2 cells (Fig. 7c). NCI-H747 cells, which harbor an activating KRAS mutation, were most affected by KRAS depletion and concomitant CIP2A knockdown did not reduce the colony formation much further (Fig. 7c). Interestingly, when all three forms of RAS were depleted, the effect on colony formation was comparable to the combination of CIP2A and KRAS (Supplementary Fig. 10). The triple RAS depletion also did not seem to synergize with CIP2A depletion (Supplementary Fig. 10). These findings suggest that either the combination of CIP2A/KRAS or HRAS/KRAS/NRAS depletion is sufficient to saturate the effect on colony formation and support the view that CIP2A and the RAS proteins regulate functionally overlapping pathways.

## Discussion

Label-free quantification methods provide an attractive option for studying cellular protein phosphorylation dynamics due to the ability to analyze large sample panels and lack of demanding labeling procedures. Label-free quantification of phosphoproteome is typically based on centering normalization of the phosphopeptide abundance; however, whether the standard normalization methods achieve sufficient accuracy has not been examined systematically in the previous literature. In this study we demonstrate that a large uni-directional change in the phosphopeptide abundance is problematic for global median centering and quantile-based normalizations. As exemplified in Figs 3 and 6c, these centering methods significantly alter the proportion of regulated phosphorylations and may therefore mislead the biological conclusions. Furthermore, quantile centering normalization is less efficient at distinguishing the sample groups despite the generally low variation between triplicates (Fig. 4). We suggest that these normalization methods should be used only in the absence of such unidirectional global phosphorylation changes. While we acknowledge the possibility that, contrary to our current results, spiking known amount of standards should help to counter the shortcomings of centering normalizations, we have found it to be too reliant on the accurate measurement of protein concentration in the cell lysates. This problem of accurate protein quantification is emphasized when the samples to be compared are heterogeneous, e.g. in clinical studies.

We have developed a novel normalization strategy, named pairwise normalization, for label-free quantitative phosphoproteomics. Its superior performance was validated by statistical methods, western blotting analysis, and bioinformatics, on the dataset obtained in this study. Pairwise normalization is based on monitoring the ratio of phosphopeptide abundances in LC-MS/MS data obtained before and after phosphopeptide enrichment (Fig. 2b). This ratio (pairwise normalization factor) is not affected by differential regulation of phosphorylations, rather it reflects variations introduced during the TiO_2_ enrichment step. Out of the two pairwise normalization methods, the one based on global centering normalization of the non-enriched digest data performed better than that based on quantile centering normalization (Fig. 5b–d). A potential explanation for the lower performance is that forcing the quantile centering normalization on the abundance distribution may introduce significant errors in the quantification of the phosphopeptides that are typically low abundance features in the non-enriched digest data.

In addition to differences observed between the normalization methods in their capacity to distinguish the sample groups, we demonstrate that the choice of normalization method influences the downstream analysis of the normalized data in terms of pathway activity predictions. In fact, the expected regulation of ERK and AKT pathways by perturbations in RAS and PP2A signaling^3^,^47^,^48^,^49^,^60^ were only observed in the global-pairwise-normalized data. Furthermore, we could demonstrate that in addition to being able to measure relevant pathway activities, the developed pairwise normalization method, when combined with pathway analysis algorithms, was able to recapitulate previously demonstrated biological synergism between RAS signaling and PP2A inhibition^12^,^14^,^17^,^18^,^61^.

Although OA was used as a potent PP2A inhibitor in this study, the OA treatment also inhibits PP4/PP6, which may have contributed to the observed results. However, many phosphosites upregulated by OA treatment, including AKT and ERK targets, were also dephosphorylated by CIP2A, suggesting PP2A involvement. The contribution of PP1/PP3 was probably minimal, e.g. Naetar *et al*. have treated cells with up to 125nM concentrations of OA without noticeable effect on the PP1 activity in subsequently purified cell lysates^18^.

Importantly, despite of wealth of functional data implicating importance of CIP2A-mediated PP2A inhibition in most of the human cancer types^12^,^13^,^62^,^63^,^64^,^65^, the phosphoprotein targets regulated by CIP2A have not been previously systematically studied. To our knowledge, this is the first systems biology analysis of CIP2A regulated phosphoproteome, and the integrated results revealed an extensive overlap with RAS regulated phosphoproteome. Additionally, it was revealed that CIP2A and KRAS exhibit a synergistic survival effect in TCGA data and their depletion resulted in synergistic reduction in colony formation. These findings suggest that PP2A inhibition and RAS cooperate in cancer progression beyond the initial transformation steps^14^. In RAS mediated transformation of immortalized cells the contribution of CIP2A has been attributed to MYC stabilization^12^,^19^; however, our results imply that the synergistic effects of CIP2A and RAS may be a consequence of a broader range of the shared signaling events, including ERK and AKT pathways.

Previous systems biology studies of RAS mediated transformation have reported prominent changes in mRNA expression^66^ and protein expression^67^,^68^,^69^ of genes associated to glycolysis and reprogramming of metabolic pathways activity. Correspondingly, in this study the Ingenuity pathway analysis of the RAS-mediated protein expression changes suggested extensive involvement in the metabolic pathways with emphasis on carbohydrate metabolism. Interestingly, analysis of phosphorylation resulted in the identification of a very divergent set of downstream effectors leading to distinctly different biological conclusions. Previous phosphoproteomics study by Sudhir *et al*. (2011) focusing on oncogenic KRAS transformed human bronchial epithelial cells identified MAPK signaling as a major component in the oncogenic KRAS downstream effectors^40^. Elevation of MAPK signaling was also observed in KRAS mutant lung adenocarcinoma cell lines. In contrast, NRAS mutant large cell carcinoma cell line exhibited lower level of MAPK signaling and prominent activity of basophilic kinases including AKT^40^. In this study, we observed downregulation of both MAPK and AKT signaling following H-, K-, and N-RAS triple knockdown.

In summary, we have developed a novel normalization strategy, pairwise normalization, for label-free quantitative phosphoproteomics. Using RAS and CIP2A depletion as well as OA treatment as model perturbations we demonstrate that pairwise normalization improves the quantitative accuracy over the conventional normalization methods tested, enabling the measurement of subtle kinase activity changes despite the global shifts introduced in phosphopeptide abundance distributions. As a potential application, ability to robustly measure kinase activity changes in clinical samples may have prognostic value and therapeutic implications e.g. in monitoring kinase inhibitor treatment efficacy and development of resistance^2^. Moreover, the commonalities in CIP2A and RAS downstream effectors, as well as their synergistic effect on cancer cell growth, strongly suggest that studying the factors regulating PP2A activity will further the understanding of the responses to therapy targeting shared downstream pathways of RAS and PP2A.

## Materials and Methods

### Cell culture

HeLa, NCI-H747, and HCA7 were obtained from ATCC (USA) and CW-2 cells from RIKEN bioresource center (Japan). DMEM was used for HeLa and HCA7. RPMI-1640 was used for NCI-H747 and CW-2. All media contained 10% FBS, 2 mM glutamine, 50 I.U./ml penicillin, and 50 μg/ml streptomycin. The cell lines were tested for mycoplasma contamination.

### Transfection

1.1 million cells were seeded on 10 cm dishes 24 hours prior to transfection. Transfection reactions were performed in 7.5 ml volume using 1.88 nmol of siRNA and 22.5 μl of Oligofectamine (Life Technologies) according to manufacturer’s instructions. RAS knockdown was performed with a cocktail siRNA targeting H-, K-, and N-RAS. The total amount of siRNA was the same (250nM) for all transfections as this did not diminish the efficiency of the triple-RAS knockdown (Supplementary Fig. 11). The siRNA sequences are listed in Supplementary Table 12. OA (Sigma-Aldrich) was added to control siRNA transfected cells at a concentration of 25nM 48 hours after transfection. Knockdowns were scaled down by a factor of 7.5 for 6-well plates.

### Cell lysates

Cells were collected by scraping in ice cold PBS 72 hours after transfection. Snap frozen cell pellets were lysed in a buffer containing 8 M urea, 50 mM Tris pH 7.5, 2 mM EGTA, 5 mM EDTA, 30 mM sodium fluoride, 60 mM B-glycerophosphate, 20 mM sodium pyrophosphate, 1 mM sodium orthovanadate, Roche complete protease inhibitor cocktail tablet, and 5 μM pepstatin A. Samples were sonicated with Bioruptor sonicator (Diagenode) at high intensity with 15 seconds pulses and intervals for 5 minutes and centrifuged at 100,000 g for 35 minutes. Supernatant was collected and protein concentration was determined by measuring the absorbance at 280 nm (10.0–17.6 mg/ml). Samples were kept at 4 degrees or on ice at all times.

### Digestion and TiO_2_ phosphopeptide enrichment

The lysates (1 mg protein) were diluted to 200 μl with a buffer containing 8 M urea and 50 mM Tris-HCl pH 8.5, after spiking in 10 μl of 1 μg/μl bovine α-casein (Sigma-Aldrich). Proteins were reduced for 1 h at 37 °C, alkylated for 30 min at R.T. in the dark, and quenched, by adding 200 mM dithiothreitol (DTT), 1 M iodoacetamide, and then 1 M DTT, respectively (each 10 μl, dissolved in the Tris/urea buffer). The proteins were digested for 18 h at 37 °C with 690 μl of 50 mM Tris-HCl pH 8.5 containing 20 μg of sequencing grade modified trypsin (Promega). After acidification with 80 μl of 10% TFA (total 1 ml), the samples were stored at −20 °C overnight or longer. Aliquots (10 μl) of the digests were desalted with a C18 microcolumn as described previously^26^,^70^ with a slight modification. Briefly, three pieces of Empore C18 disk (3M) were packed into a 200-μl pipette tip, followed by pretreatment with acetonitrile (ACN) and 0.1% formic acid (FA), sample loading, 3 times washing with 0.1% FA, and then elution with 0.1% FA, 80% ACN (each 50 μl loaded by gentle air pressure). The eluents were evaporated to dryness. For LC-MS/MS analysis, the samples (non-enriched digests) were reconstituted in 50 μl of 0.1% FA, of which 6 μl was transferred into an LC sample vial for 5 μl injection.

The remaining digests (990 μl) were desalted as well, with some modifications. Briefly, an Empore C18-SD 10mm/6mL cartridge (3M) was pretreated with ACN and 0.1% TFA, followed by sample loading (repeated once again), 3 times washing with 0.1% TFA, and then elution with 6% TFA, 80% ACN (each 1 ml loaded by gentle air pressure). Phosphopeptides were enriched by TiO_2_ affinity chromatography as described by Imanishi *et al*.^26^ with some modifications: 100 μl of 50 mg/ml Sachtopore-NP TiO_2_ beads (20 μm, 300 Å; ZirChrom) prewashed with 10% TFA and ACN was packed into a 200-μl tip (with three pieces of GF/C disk (Whatman) as a column frit), followed by pretreatment with the TFA/ACN solution, loading whole sample, washing with TFA/ACN twice and 0.1% TFA twice, and then elution with 5% NH_4_OH (each 200 μl loaded by gentle air pressure). Immediately, the eluents were acidified with 400 μl of 10% FA, desalted with the C18 microcolumn, and then evaporated (the immediate desalting prevents induced methionine oxidation^26^). For LC-MS/MS analysis, the samples (TiO_2_-enriched phosphopeptides) were reconstituted in 11 μl of 0.1% FA, of which 5.5 μl was transferred into an LC sample vial for 5 μl injection.

### Mass spectrometry, identification, and localization

LC-MS/MS analysis was performed using an EASY-nLC 1000 nanoflow LC instrument coupled to a Q Exactive quadrupole-orbitrap mass spectrometer (Thermo Fisher Scientific). Data of the TiO_2_-enriched and non-enriched samples were searched with Mascot (v2.4.1) via Proteome Discoverer (v1.4.0.288, Thermo Fisher Scientific), against a concatenated forward-reverse SwissProt database (v2012_04, *Homo sapiens*) supplemented with common contaminants (total 40,678 protein sequences). For phosphorylation site localization, phosphoRS (v3.0, the neutral loss option disabled) was enabled. Also, the data of the TiO_2_-enriched samples were searched against an in-house made spectral library of simulated phosphopeptides (14,761 spectra for 3,208 peptide sequences) with SpectraST via Proteome Discoverer (SimSpectraST searching^46^). The two search results were merged into an identification result. More details are described in Supplementary methods.

### Label-free quantification

Label-free quantification was performed using Progenesis LC-MS (v4.1). The TiO_2_-enriched and non-enriched samples were processed separately. All the chromatographic data were aligned automatically and further adjusted manually. Peptide ion features were detected in the automatic mode with the highest sensitivity. The features were assigned by importing the xlsx identification file, followed by applying a 5-ppm mass tolerance filter. For the non-enriched samples, all the features assigned to human peptides were used for global median centering of abundance ratios (global centering normalization). Protein abundance was quantified based on the sum of ion abundances of peptides unique to a protein. For the TiO_2_-enriched samples, in addition to the global centering normalization based on all the human phosphopeptide features, normalization to the median ratio of casein phosphopeptide features was performed. Quantile-based normalization was performed on the abundances of human peptide features (quantile centering normalization) using R-package preprocessCore (v1.26.1). After annotating phosphorylation sites on proteins, abundance of phosphosites (or their combinations) was quantified based on the sum of ion abundances of phosphopeptide variants (i.e. different charge, missed cleavage, oxidation, and/or acetylation states). When a phosphosite was assigned to both high and low localization confidence features, only the former was taken into account.

### Pairwise normalization factors

Phosphopeptides shared by both the TiO_2_-enriched digests and non-enriched samples were used for the pairwise normalization (refer to Fig. 2b). After the global centering normalization, abundances of phosphopeptide ion features with the identical sequences and modifications were summed up, separately in the TiO_2_ and digest datasets. Methionine-containing phosphopeptides were excluded due to the possible oxidation during the sample preparation, resulting in 52 unique phosphopeptides quantified in both the samples. An abundance ratio (digest/TiO_2_) was calculated for each phosphopeptide, followed by normalization to one of the 15 biological samples. If the variation in the normalized abundance ratios between the biological samples exceeded a threshold value of 16.4 (determined by box plot analysis, see Supplementary Fig. 3), the phosphopeptide was removed as an outlier. The median of the normalized abundance ratios was used as the pairwise normalization factor, by which all the phosphopeptide abundances in the TiO_2_ dataset were multiplied. The factor was calculated based on the quantile-centering-normalized non-enriched digest data as well.

### Western blotting

Antibodies for p-S6 (2211S), p-STAT3 (9134L), p-ERK (4370), and p-GSK3β (9336) were purchased from Cell Signaling, the p-MYC antibody (ab78318) from Abcam, the GAPDH antibody (5G4–6C5) from Hytest, and β-actin antibody (A5316) from Sigma-Aldrich. The antibody for p-vimentin was a generous gift from professor John Eriksson (Åbo Akademi University, Turku, Finland). Western blot band intensities were quantified with ChemiDoc MP imaging system and Image Lab 4.0.1 software (Bio-Rad). Multiple exposures were used for quantitation when necessary. The band intensities were normalized between exposures to the average of bands quantitated from both exposures. We used GAPDH and ACTB as loading controls for western blotting. However, the abundance ratio of these two commonly used loading control proteins varied between the treatments used in this study (Supplementary Fig. 12), indicating the variation in their abundance. Therefore, GAPDH and ACTB abundances were adjusted to the LC-MS/MS data that were used to determine the respective pairwise normalization factors, which improved the average correlation between western blot and the pairwise-normalized data (Supplementary Table 9).

### Colony formation assay

For colony formation assays, 2000 HeLa, HCA7 or NCI-H747 cells and 4000 CW-2 cells were seeded on 6-well plate wells 72h after transfection. HCA7 colonies were grown for 8 days, HeLa colonies for 10 days, and NCI-H747 and CW-2 for 11 days. Cells were fixed with ice cold methanol and stained with 0.1% crystal violet in 10% ethanol. After washing, colonies were scanned at 2400 dpi and colony area and intensity were quantified with ImageJ software using ColonyArea plugin^71^.

### Bioinformatics

Ingenuity Pathway Analysis was performed on the 2014 spring release version (QIAGEN). The kinase target predictions were performed using NetworKIN 3.0^55^ and GPS 2.0^56^ software. The target site data in the PhosphoSitePlus database^57^ was downloaded in May 2014. TCGA PANCAN gene expression and mutation data sets were obtained via UCSC Cancer Genomics Browser^72^,^73^ in June 2014. The 5 year survival rates for different cancer types were obtained from the SEER data collected during the years 1975–2011^74^. Hierarchical clustering is described in Supplementary methods.

### Statistics

All t-tests were performed as two-tailed and assuming equal variance. Levene’s test was used for comparing the standard deviations of peptide intensity distributions (Supplementary fig. 4b) and confirming the equality of variance in colony formation assay results (Fig. 7b). Survival distributions were compared using the Log-rank test. Shapiro-Wilk test was used for assessing the normality of western blot correlation data (Supplementary table 8) and colony formation assay data (Fig. 7b). Due to the large sample size, the approximate normality of the phosphopeptide feature abundance distribution was visually assayed (Supplementary fig. 4a). Statistical calculations were performed with JMP 10.0.0 software (SAS Institue inc.).

### Data deposition

Raw mass spectrometry data, protein sequence databases, spectral libraries, search results, and a peptide/protein identification list have been deposited to the ProteomeXchange Consortium (http://proteomecentral.proteomexchange.org)^75^ via the PRIDE partner repository with the dataset identifier PXD001374 (to access the data for the review purpose: http://tinyurl.com/k47j47t, reviewer account username: reviewer90019@ebi.ac.uk, password: qmEA0bnn). For viewing annotated MS/MS spectra in the Proteome Discoverer results (msf files), a free viewer is available from the Thermo Omics Software Portal (http://portal.thermo-brims.com).

## Additional Information

**How to cite this article**: Kauko, O. *et al*. Label-free quantitative phosphoproteomics with novel pairwise abundance normalization reveals synergistic RAS and CIP2A signaling. *Sci. Rep*. **5**, 13099; doi: 10.1038/srep13099 (2015).

## Supplementary Material

Supplementary Information

Supplementary Table S1

Supplementary Table S2

Supplementary Table S3

Supplementary Table S4

Supplementary Table S5

Supplementary Table S6

Supplementary Table S7

Supplementary Table S8

Supplementary Table S9

Supplementary Table S10

Supplementary Table S11

Supplementary Table S12

Supplementary Table S13

## Figures and Tables

**Table 1:** Identification and quantification of HeLa proteins and phosphorylations.

	HeLaa,^b^		Alpha-casein^a^ (spiked protein)
	All	High confidence site (1% FLR)	
TiO_2_-enriched samples			
Phosphopeptide spectral matches (0.18% FDR)	41605	29029	1677
Identified phosphopeptides	4519	2740	37
Quantified phosphopeptide features	4026	2935	73
Quantified phopshopeptides	3073	2217	27
Quantified phosphosite combinations	2621	1873	
Phosphosites	2911	2051	
Phosphoproteins	1255	1067	
Non-enriched digests			
Peptide spectral matches (0.15% FDR)	176681		750
Identified peptides	16344		31
Identified phosphopeptides	89	51	8
Quantified features	16922		60
Quantified peptides	14015		31
Quantified phosphopeptides	68	43	8
Quantified proteins	2567		
>1 unique peptides quantified	1724		

^a^A peptide with and without methionine oxidation was counted as 1.

^b^Phosphosites shared by different proteins were counted repeatedly, i.e. those were redundant.
