# Supplementary material for: Label-free quantitative phosphoproteomics with novel pairwise abundance normalization reveals synergistic RAS and CIP2A signaling
Source: Sci Rep. 2015 Aug 17;5:13099. doi: 10.1038/srep13099 (PMC4642524; doi:10.1038/srep13099)
Supplement: Supplementary Information [file srep13099-s1.pdf]

Supplementary material

Label-free quantitative phosphoproteomics with novel pairwise abundance normalization reveals synergistic RAS and CIP2A signaling

Otto Kauko, Teemu Daniel Laajala, Mikael Jumppanen, Petteri Hintsanen, Veronika Suni, Pekka Haapaniemi, Garry Corthals, Tero Aittokallio, Jukka Westermarck, and Susumu Y. Imanishi

Supplementary methods

Mass spectrometry

LC-MS/MS analysis was performed using an EASY-nLC 1000 nanoflow LC instrument coupled to a Q Exactive quadrupole-orbitrap mass spectrometer (Thermo Fisher Scientific). A 100 µm x 2 cm trap column and a 75 µm x 15 cm analytical column were in-house packed with Magic C18AQ resin (200 Å, 5 µm; Michrom Bioresources). The mobile phases were 2% ACN, 0.2% FA (A) and 95% ACN, 0.2% FA (B). LC gradient elution condition was initially 2% B to 20% B (70 min), 40% B (100 min), and 100% B (105-110 min), with a flow rate of 300 nl/min. Data dependent acquisition was performed in positive ion mode. MS spectra were acquired from *m/z* 300 to *m/z* 2000 with a resolution of 70,000 at *m/z* 200, a target value of 1,000,000 ions, and a maximal injection time of 120 ms, in profile mode. The 10 most abundant ions of which charge states were 2+ or higher were selected for subsequent fragmentation (higher energy collisional dissociation, HCD) with normalized

collision energy of 30, and MS/MS spectra were acquired with a resolution of 17,500 at *m/z* 200, a target value of 50,000 ions, a maximal injection time of 250 ms, and the lowest mass fixed at *m/z* 100, in profile mode. Dynamic exclusion duration was 30 s. The lock-mass option was used (*m/z* 445.12003).

Enzymatic dephosphorylation

After LC-MS/MS analysis of the TiO_2-enriched samples, the samples remaining in the vials (<0.5 µl each) were collected in an Eppendorf tube and evaporated. Enzymatic dephosphorylation was performed according to Imanishi et al. [26]. Briefly, the collected sample was reconstituted in 10 µl of 100 mM NH_4HCO_3, followed by dephosphorylation for 1 h at 37 °C with 10 µl of 100 mM NH_4HCO_3 containing 0.1 U/µl EIA grade calf intestinal alkaline phosphatase (Roche), acidification with 80 µl of 0.1% FA, desalting with the C18 microcolumn, and then evaporation. For the analysis, the dephosphorylated sample, as well as those obtained from 5 other experiments (on PP2A-regulated phosphoproteomes in HeLa cells, which will be reported elsewhere; totally collected from >90 vials), was reconstituted in 5.5 µl of 0.1% FA, of which 5 µl was loaded to LC-MS/MS.

Identification by database and spectral library searching

LC-MS/MS data of the TiO_2-enriched and non-enriched samples were searched with Mascot (v2.4.1) via Proteome Discoverer (v1.4.0.288, Thermo Fisher Scientific), against a

concatenated forward-reverse SwissProt database (v2012_04, *Homo sapiens*) supplemented with common contaminants (total 40,678 sequences). The search criteria were: trypsin as an enzyme; two missed cleavage sites allowed; carbamidomethylation of cysteine as a fixed modification; oxidation of methionine, phosphorylation of serine/threonine/tyrosine and acetylation of protein N-terminus as variable modifications; peptide mass tolerance 5 ppm; and MS/MS ion tolerance 0.02 Da. For phosphorylation site localization, phosphoRS (v3.0, the neutral loss option disabled) was enabled. In parallel, peak lists (mgf files) for the TiO_2-enriched samples were exported.

A spectral library of simulated phosphopeptides was created as described by Suni et al. [46]. Briefly, MS/MS spectra of all possible isoforms of singly phosphorylated peptides were simulated using SimPhospho program based on 6 LC-MS/MS data of the dephosphorylated peptides, where PeptideProphet probability ≥0.95, Mascot expectation value ≤0.05, and delta score ≥10 were applied for Mascot search as a cutoff. A consensus spectral library (SimPP2A) was built using SpectraST (version 4.0, released beta) [76], which consists of 14,761 spectra for 3,208 peptide sequences. SpectraST searching against the SimPP2A library (SimSpectraST searching) was performed for the TiO_2-enriched samples via Proteome Discoverer by submitting the mgf files. The SwissProt database without the reversed sequences was used, and peptide mass tolerance was set to 3 Da. For estimating probability, Percolator was enabled. The search was repeated against the library supplemented with decoy entries, using the stand-alone SpectraST (after adjusting the

mgf file format) to obtain its F-value and recalculated deltaDot score that are required for phosphorylation site localization.

All the Mascot and SimSpectraST results were merged on Proteome Discoverer. An xlsx file was exported after applying Mascot expectation value ≤0.05, Percolator PEP ≤0.05, minimum 7 amino acid length, search engine rank 1, and protein grouping. As cutoffs for 1% FLR (i.e. high confidence phosphorylation sites), phosphoRS probability ≥99.3% was used, while SpectraST (stand-alone) F-value ≥0.49 and recalculated deltaDot score ≥0.005 were applied only when a singly phosphorylated peptide contained >1 Ser/Thr/Tyr in its sequence [46]. Based on the localization confidence (high or low), a new score was given for each phosphopeptide spectral match. When Mascot and SimSpectraST searches disagreed on high confidence phosphosites, those were considered as low confidence. The scoring scheme is summarized in Supplementary Table 13. An FDR was estimated using the target-decoy strategy at a (phospho)peptide spectral match level, i.e. 2 x decoy matches / total matches.

Hierarchical clustering

Unsupervised clustering of the 15 samples from 5 distinct sample groups was done using different clustering strategies and distance metrics. A distance matrix was computed for the samples after log-transformation using one of the chosen similarity metrics: Euclidean distance, Manhattan distance, maximum distance, or Pearson correlation. Hierarchical

clustering was then performed on the 15×15 dissimilarity matrices using one of the following agglomerative hierarchical cluster building strategies: complete linkage, single linkage, average linkage, or Ward's minimum variance. Thus, for each of the 5 normalization methods, a total of 16 different combinations were run for building the clusters. A representative example of the clustering solutions (Euclidean distance metric with Ward's minimum variance cluster building strategy for the global pairwise normalized data) is presented in Fig. 4a.

Adjusted Rand index was used to evaluate agreement between the clustering solutions and the original sample labels using the R-package mclust (v4.3). The resulting hierarchical clustering trees were progressively height cut to create 1,2,3,…,14,15 clusters of samples. These cluster labels were then compared to the original sample labels using the adjusted Rand index. Fig. 4a shows example height cuts for obtaining solutions with 2,3,6 clusters (horizontal lines). The resulting solutions for these example height cuts are visualized in Fig. 4b. These progressive height cuts resulted in Rand index curves similar to the ones shown in Fig. 4c. In order to obtain a single comparable value for each of the methods, AUC was computed for these curves, with larger AUC values indicating better performance.

Supplementary references

76. Lam, H. et al. Development and validation of a spectral library searching method for peptide identification from MS/MS. *Proteomics* **7**, 655-667 (2007).

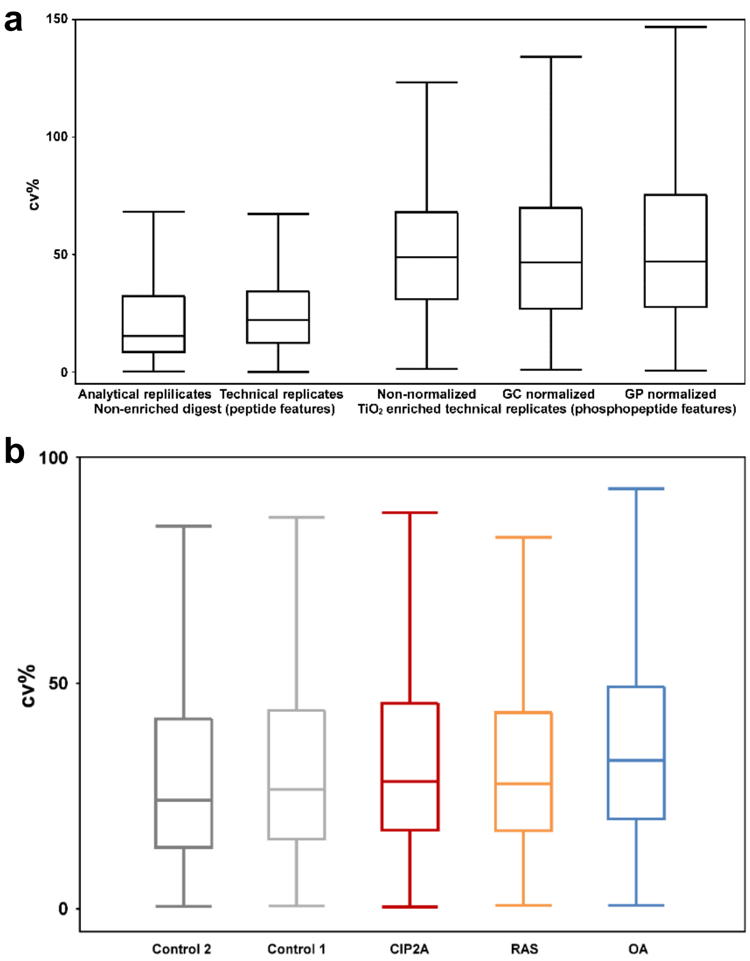

Supplementary figure 1

Supplementary figure 1. The effect of sample preparation steps on the variation between triplicate measurements

(a) Coefficient of variation (CV) was calculated for all quantified peptide features in triplicate analyses after global centering normalization. The distribution of CV values is shown as a box plot. The whiskers represent 1.5x interquartile range. The distributions correspond to analytical replicates of the same non-enriched control sample (peptide features) as well as technical replicates of sample preparation with and withoutTiO_2 enrichment (phosphopeptide and peptide features, respectively). The TiO_2 enrichment is a major contributor to the observed variation. The global centering and global pairwise normalizations had similar effect on the CV distributions, resulting in 2.17 and 1.87 improvement in median CV, respectively. (b) The CV distributions of the TiO_2 enriched samples with the different treatments are shown (phosphopeptide features).

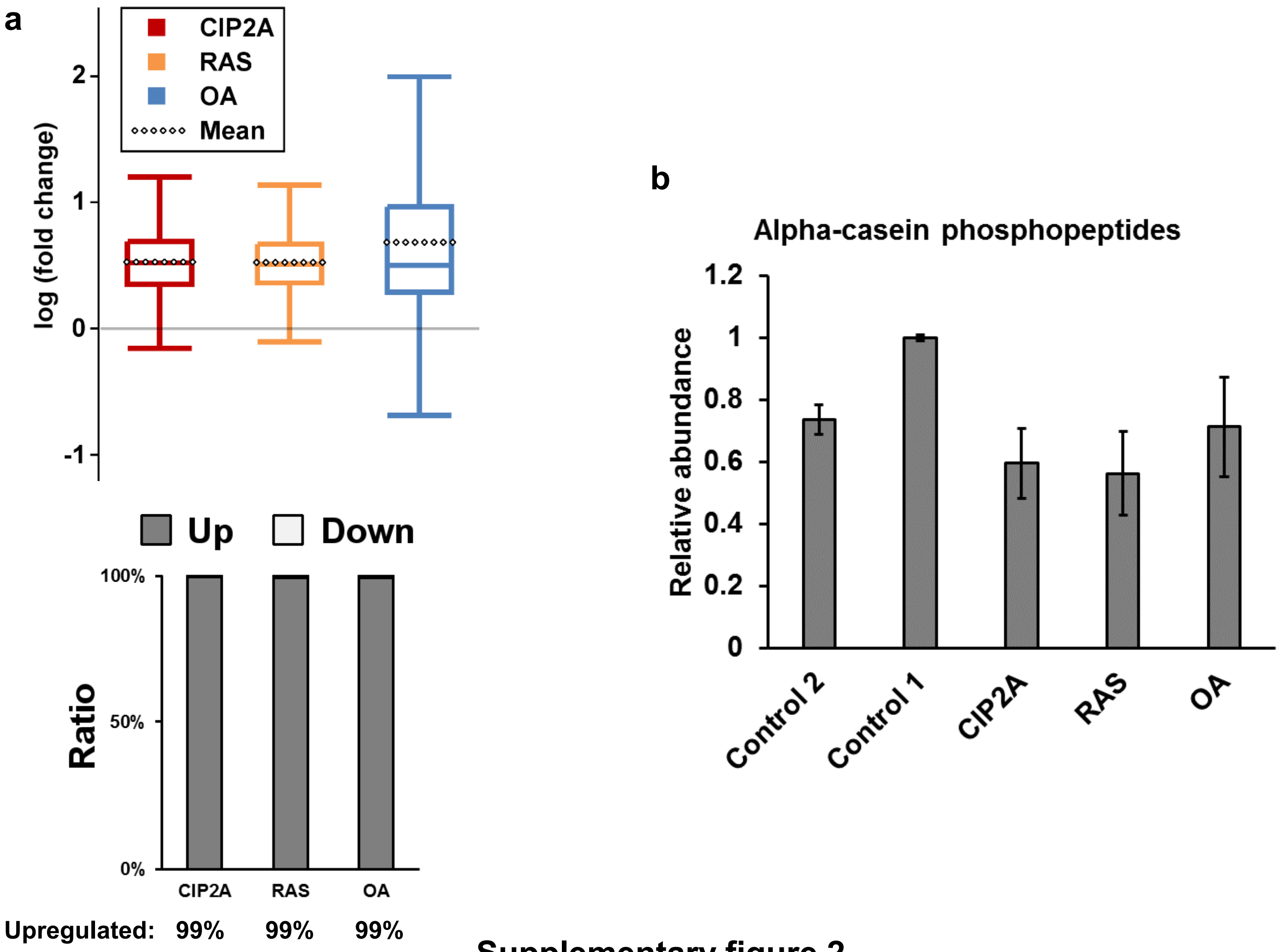

Supplementary figure 2

Supplementary figure 2. Spiked-in alpha-casein normalization

(a) Fold change distribution of phosphorylations (upper panel) and the ratio of up- and down-regulated phosphosites (lower panel, differentially regulated phosphosites compared to the control 1 samples; ttest, $p<0.01$) following the spiked-in alpha-casein normalization. (b) Comparison of phosphopeptide abundance of spiked-in alpha-casein between samples. The sum of quantified alpha-casein phosphopeptide abundances in the nonenriched digests after global centering normalization is shown. The error bars represent standard error of the mean.

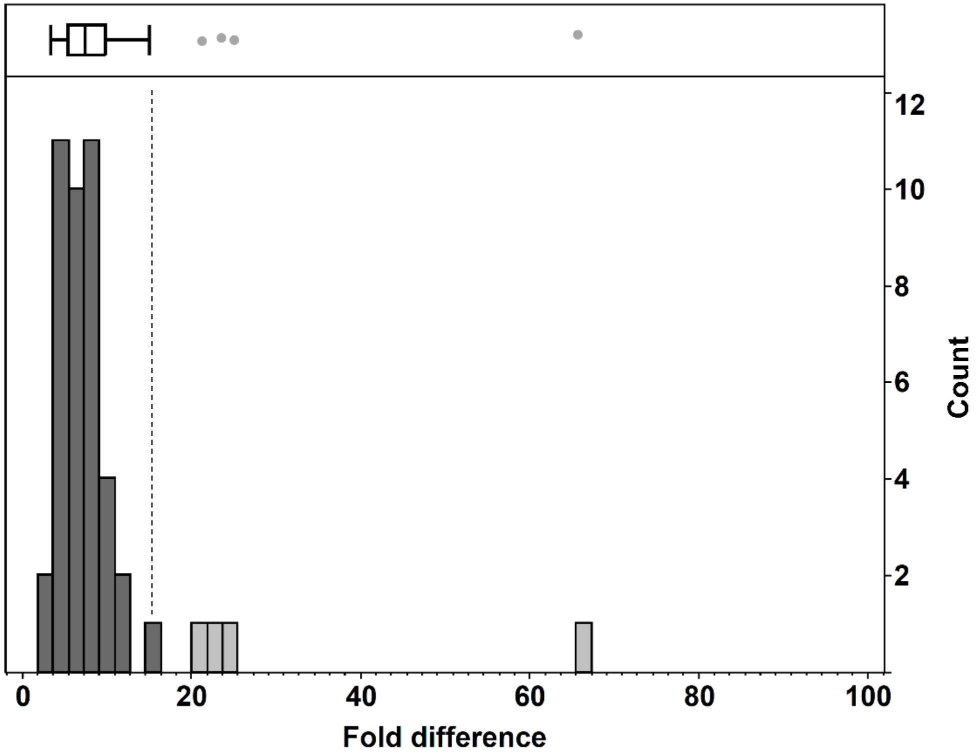

Supplementary figure 3

Supplementary figure 3. Exclusion criteria for phosphopeptides used for calculating the pairwise normalization factor. For phosphopeptides quantified both in the non-enriched digests and TiO_2-enriched samples (52 unique phosphopeptides), digest/TiO_2 abundance ratios were calculated for each phosphopeptide after global centering normalization, and these ratios were normalized to one of the 15 biological samples. A maximum fold difference in the abundance ratios between the samples was calculated for each phosphopeptide and plotted in the figure. The dashed line indicates the selected cutoff for outliers, which corresponds to a boxplot analysis with whisker representing 1.5x interquartile range (maximum fold difference of 16.4). Three outliers are outside the figure scale and for peptides were excluded due to missing data for some samples.

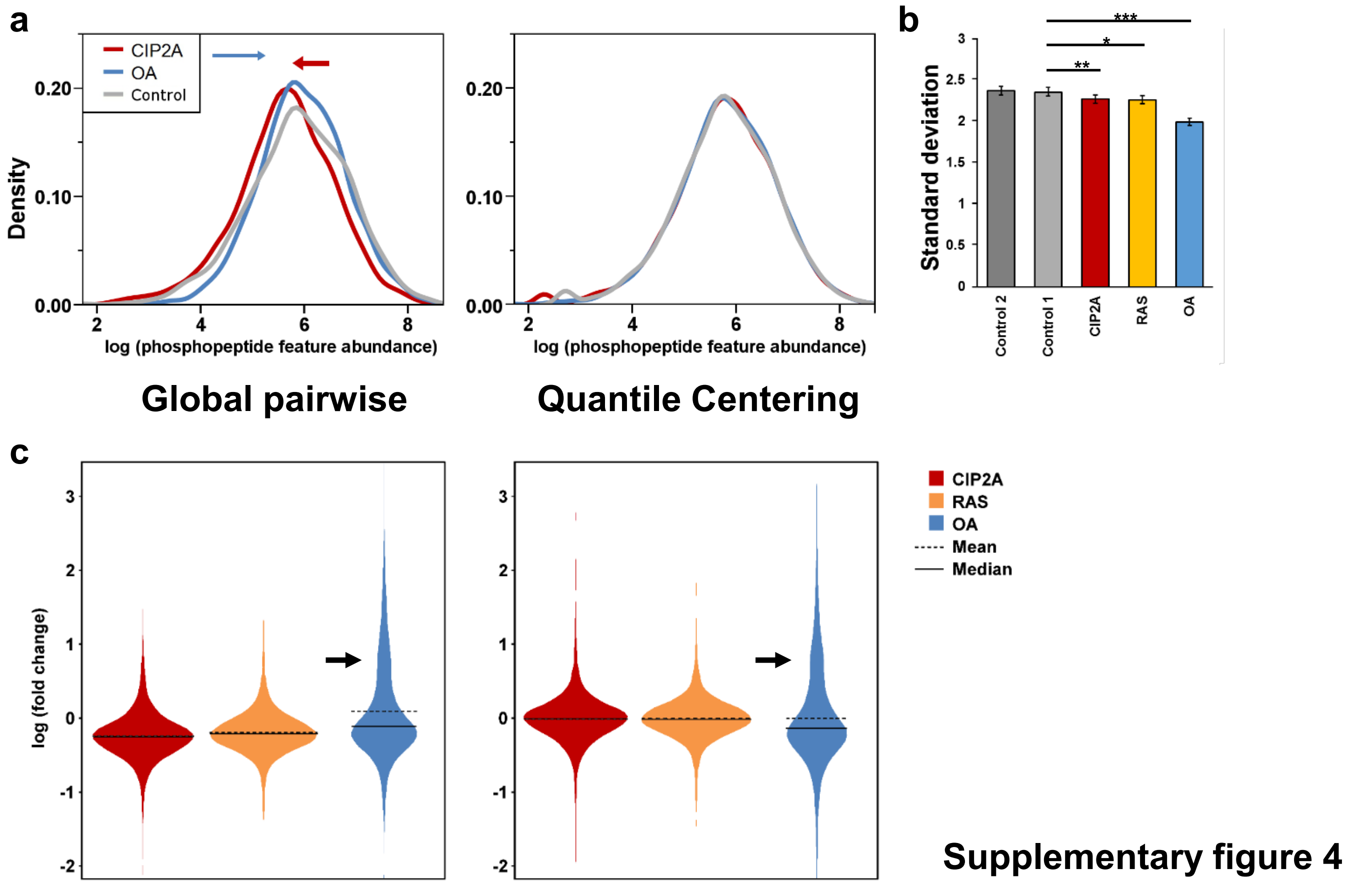

Supplementary figure 4. Effect of CIP2A and OA treatments on phosphopeptide abundance distribution.
(a) Abundance distributions of phosphopeptide ion features (the average of triplicates, log-transformed). The CIP2A, OA and control 1 data normalized either with the global pairwise or quantile centering methods are shown as representative examples. The distribution profile was altered with CIP2A depletion and OA treatment in the global pairwise normalized data (this was also seen in the data normalized with the casein, global centering, and quantile pairwise methods). CIP2A depletion was characterized by a shift of high abundance features towards the median abundance and OA treatment by several fold upregulation of low abundance features (as indicated with arrows). However, these characteristic changes were not observed in the quantile centered distribution. (b) The standard deviations of the abundance distributions were calculated from the global pairwise normalized data. The error bars represent 95% confidence intervals. The standard deviations were significantly reduced in the CIP2A, RAS and OA data, while those of the controls 1 and 2 were apparently similar (Levene's test *p=0.0282, **p=0.0037, and ***p=0.0001). (c) The fold change distributions of the phosphopeptide features were compared between the CIP2A, RAS and OA samples (compared to the control 1 samples, log-transformed). The OA treatment resulted in a long tail in the distribution (indicated by an arrow) and in the increased mean value compared to the median, both in the global pairwise and quantile centering methods (also seen in the data with the other three normalizations).

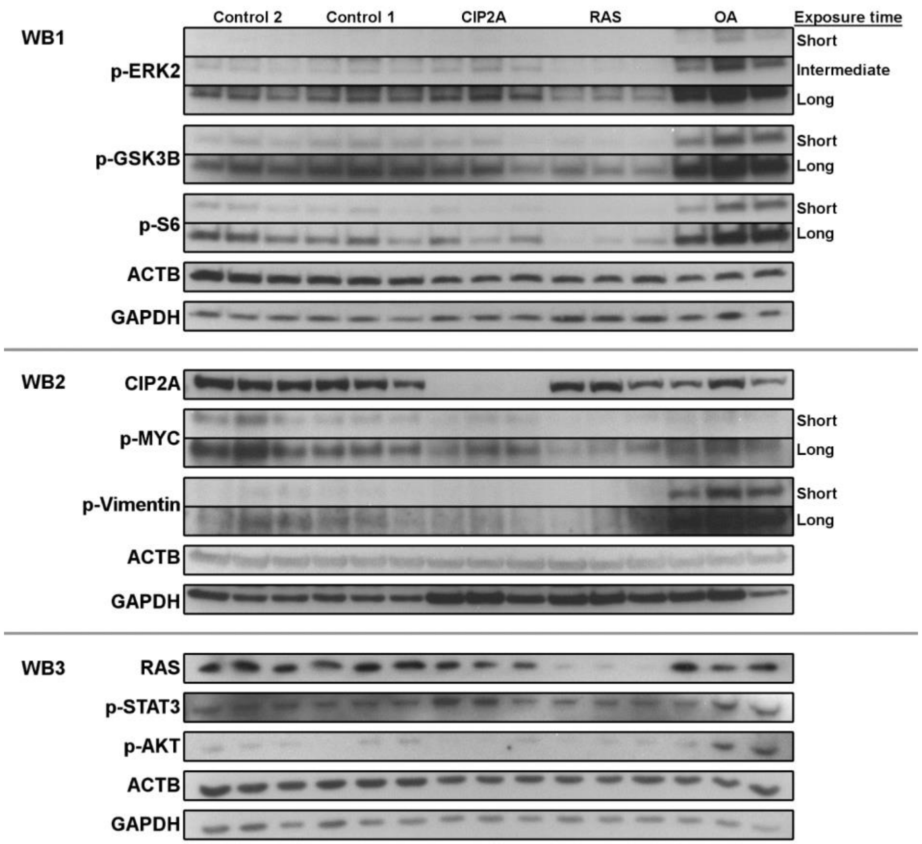

Supplementary figure 5

Supplementary figure 5. Western blots with different exposure times used for quantification.
Multiple exposure times were used when the difference between samples exceeded the linear range of western blot quantification. The band intensities were normalized between different exposure times using the average of all the intensities quantified in both exposures. The results of quantification are presented in Figure 5b and Supplementary table 8.

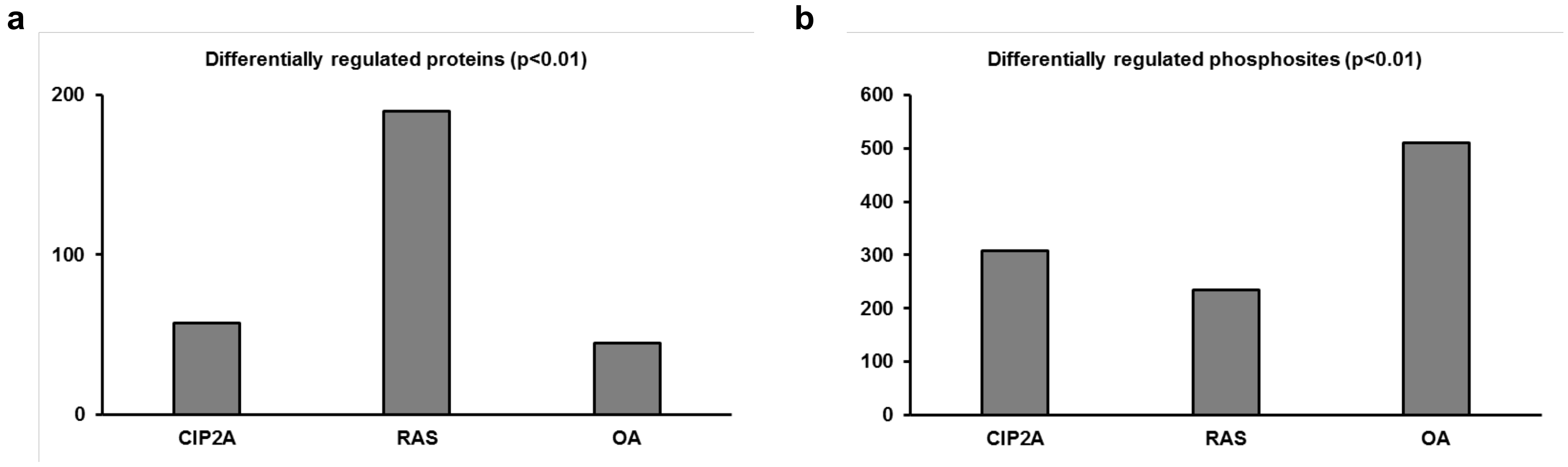

Supplementary figure 6

Supplementary figure 6. Number of differentially regulated proteins and phosphosites with the different treatments.
(a) Differentially regulated proteins with the different treatments (compared to the control 1; t-test, p<0.01) were observed in the non-enriched digests after global centering normalization. The numbers of those proteins are shown in the figure.
(b) Differentially regulated phosphosites were observed as well, in the TiO2-enriched samples after global pairwise normalization.

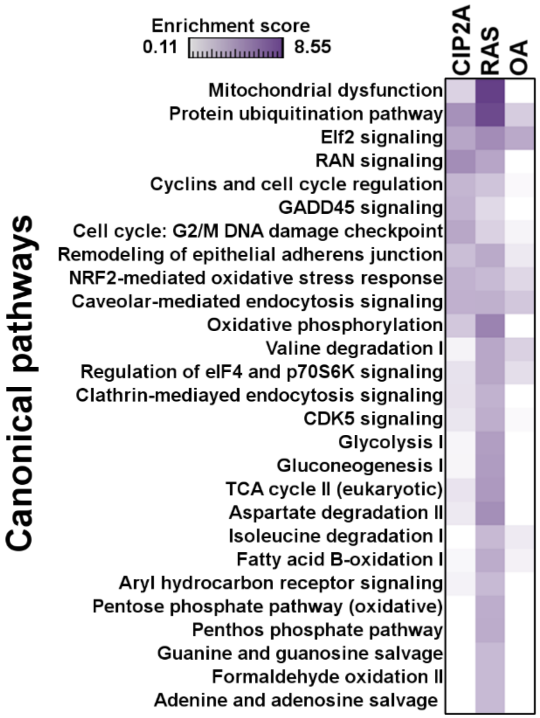

Supplementary figure 7

Supplementary figure 7. Pathway analysis based on the protein expression changes.
As shown in Figure 6a, Ingenuity Pathway Analysis was performed for differentially regulated proteins (the core analysis; t-test, $p<0.05$) after global centering normalization of the non-enriched digests data followed by the comparison analysis of the CIP2A, RAS, and OA samples. In this figure, the top hits from the comparison analysis category “canonical pathways” are shown. Multiple pathways regulated uniquely by RAS are involved in carbohydrate metabolism and other metabolic processes.

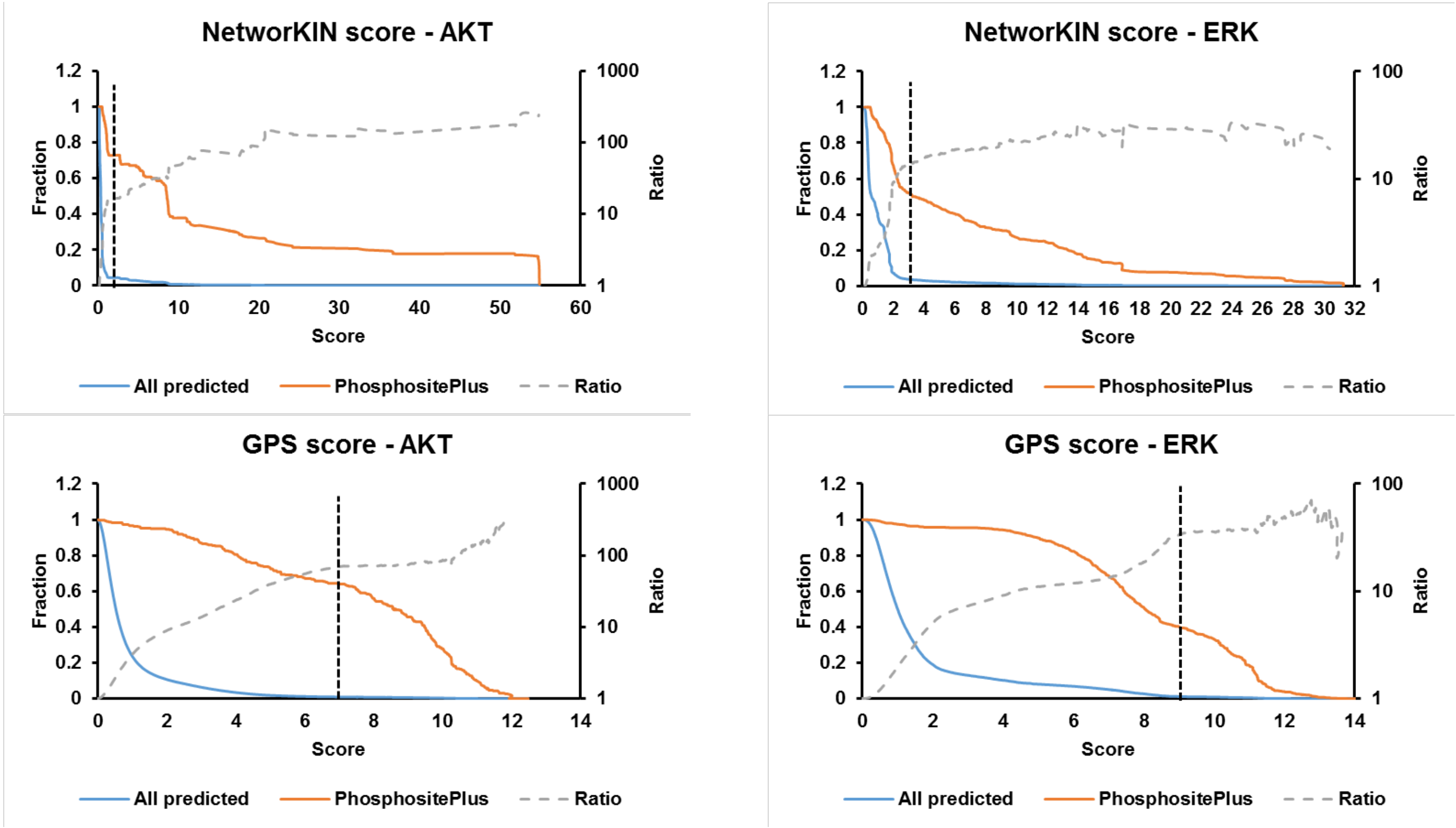

Supplementary figure 8

Supplementary figure 8. Cutoff scores for kinase target prediction tools, NetworKIN and GPS.
Phosphorylation sites quantified in this study were analyzed for potential AKT and ERK target sites using NetworKIN 3.0 and GPS 2.0 tools. In order to optimize cutoffs for their prediction scores, the AKT and ERK target sites curated from literature into the PhosphositePlus database were also analyzed and the obtained scores were used as reference data. The fractions of the PhopsphositePlus sites and the predicted sites quantified in this study were plotted as a function of prediction score. The fraction ratios of the PhosphositePlus sites to predicted target sites were examined in these plots, and score thresholds were set to values where these ratios started plateauing (indicated by vertical dashed lines). This resulted in cutoffs stricter than the preset high threshold in GPS (NetworKIN AKT cutoff 2, NetworKIN ERK cutoff 3, GPS AKT cutoff 7, GPS ERK cutoff 9).

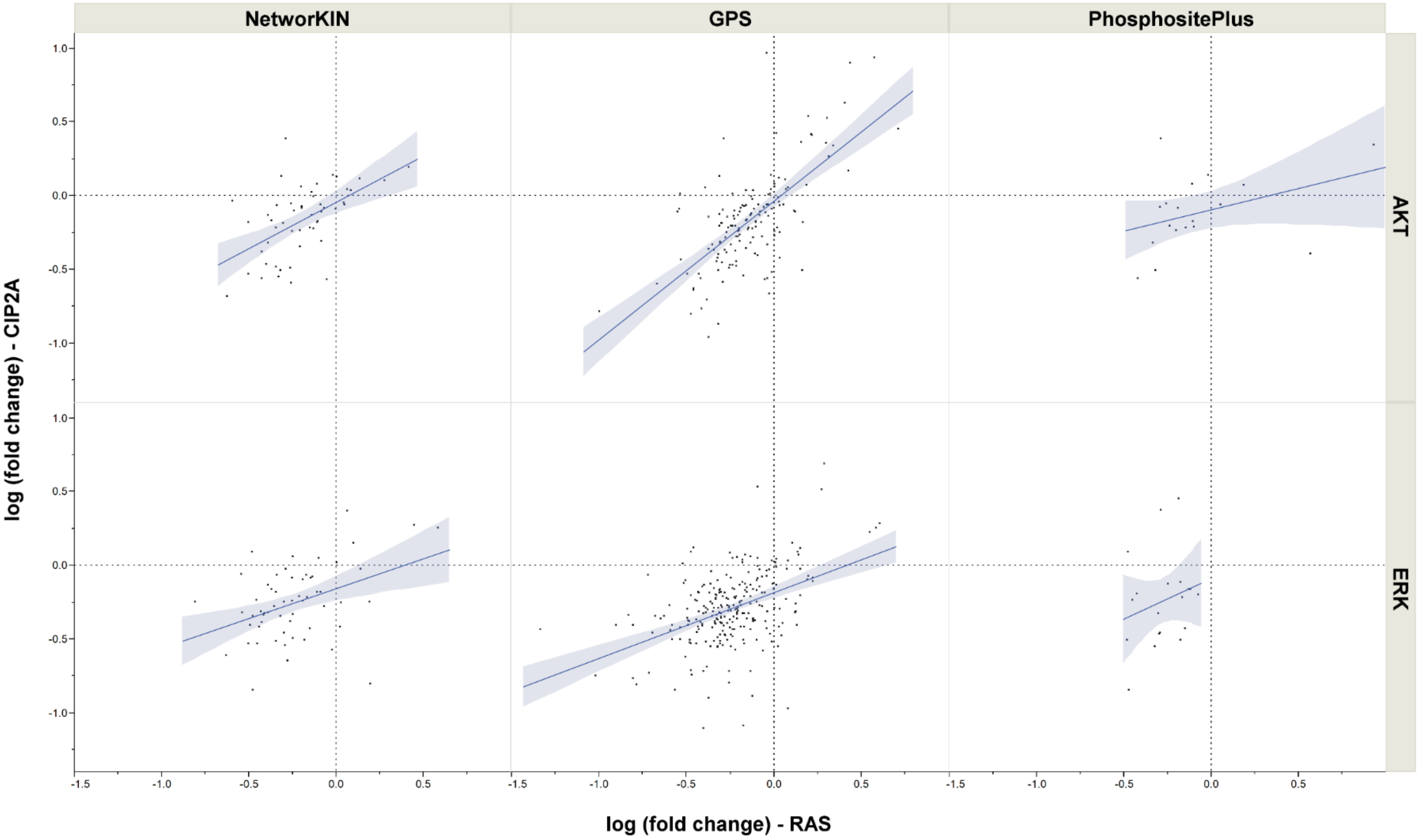

Supplementary figure 9

Supplementary figure 9. Correlation of AKT and ERK target phosphorylations between the CIP2A and RAS samples. The AKT and ERK target phosphosites were quantified after global pairwise normalization of the TiO2-enriched sample data, and the fold changes were calculated compared to the control 1 samples (see also Figure 6c) The phosphorylation changes in the CIP2A samples are assigned to the vertical axis and those in the RAS samples to the horizontal axis. The linear fit with the 95% confidence intervals is indicated as the shaded area. Positive correlation was observed in the AKT and ERK targets between the CIP2A and RAS samples (see also Supplementary table 10).

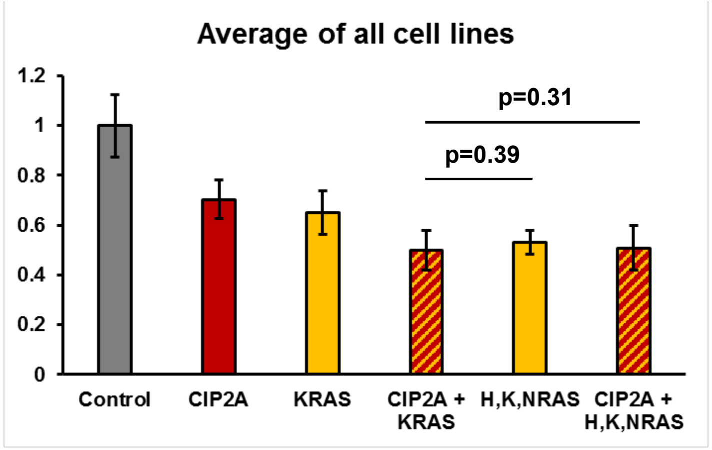

Supplementary figure 10

Supplementary figure 10. Effect of triple RAS knockdown on colony formation.
The colony formation results are shown as similar in Figure 7b, but here including the triple RAS and CIP2A + triple RAS knockdowns. Assay was performed 3 times using different siRNAs for CIP2A in HeLa, CW-2, HCA7, and NCI H747 cell lines. The average results are shown. The effect of the triple RAS knockdown was comparable to that of the combination of CIP2A and KRAS, and the addition of CIP2A knockdown did not result in noticeable decrease compared to the triple RAS knockdown in colony formation ability. The error bars represent standard error of the mean.

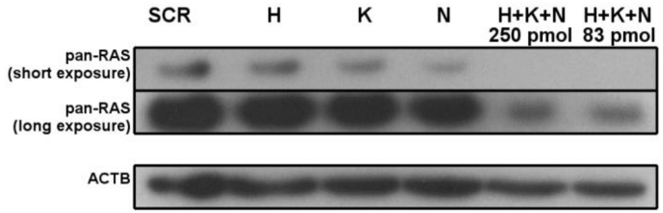

Supplementary figure 11

Supplementary figure 11. Efficiency of triple RAS knockdown with a reduced concentration of siRNAs.
RAS proteins were depleted using 250 pmol of siRNAs of the indicated isoforms in 1ml transfection in one 6-well plate well. The triple knockdown efficiency with 250 pmol of siRNAs for the three isoforms (i.e. total 750 pmol) was comparable to that with 250 pmol in total. The knockdown efficiency was evaluated using pan-RAS antibody.

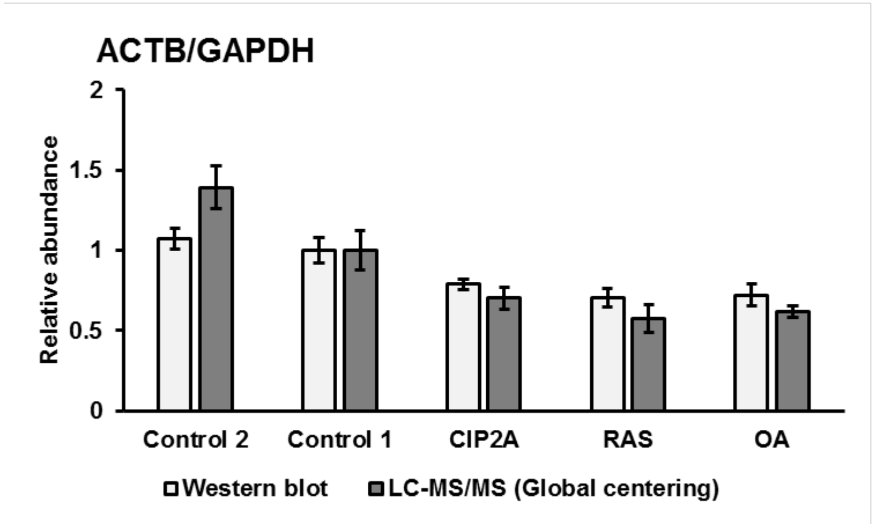

Supplementary figure 12

Supplementary figure 12. The sample treatments altered the ACTB/GAPDH abundance ratio.
The abundance ratio of loading control proteins, ACTB and GAPDH, was determined from the LC-MS/MS data of the non-enriched digests after global centering normalization, and from the average of 3 western blots (see Suplemementary figure 5 and Supplementary table 8). With the exception of the control 2 samples, the western blots and LC-MS/MS data exhibited a highly similar trend. The RAS and CIP2A depletions as well as the OA treatment resulted in lower ACTB/GAPDH ratio in both the quantifications. The error bars represent standard error of the mean.
